# Treatment approaches and costs associated with diabetes clinical metrics as measured by Healthcare Effectiveness Data and Information Set (HEDIS)

**DOI:** 10.1186/s12913-024-10745-2

**Published:** 2024-03-26

**Authors:** Jamil Alkhaddo, Jillian M. Rung, Ameer Khowaja, Yue Yin, Shannon B. Richards, Charlotte Drury-Gworek, Samina Afreen, Caitlan Rossi, Susan Manzi

**Affiliations:** 1 Allegheny Health Network, Division of Endocrinology, Pittsburgh, PA USA; 2 Highmark Health Enterprise Data & Analytics, Pittsburgh, PA USA; 3 Northeast Endocrinology Associates, San Antonio, TX USA; 4 Allegheny-Singer Research Institute, Pittsburgh, PA USA; 5 Division of Endocrinology, University of Virginia, Charlottesville, VA USA; 6 Allegheny Health Network Medicine Institute, Pittsburgh, PA USA

**Keywords:** Diabetes, Healthcare Effectiveness Data and Information Set (HEDIS), Certified diabetes care and education specialist (CDCES), Electronic consults (E-consults), Patients-to-providers

## Abstract

**Background:**

The clinical outcomes of diabetes can be influenced by primary care providers’ (PCP) treatment approaches. This study explores the association between PCP approaches to management and performance measured by established diabetes metrics and related costs.

**Methods:**

In phase one, Electronic Medical Records were used to extract diabetes related metrics using Healthcare Effectiveness Data and Information Set (HEDIS), for patients with diabetes who had office visits to 44 PCP practices from April 2019 to March 2020. Using those metrics and scoring system, PCP practices were ranked and then categorized into high- and low-performing groups (top and bottom 25%, *n* = 11 each), with a total of 19,059 clinic visits by patients with a diagnosis of diabetes. Then extensive analysis was performed to evaluate a correlation between treatment approaches and diabetes outcomes across the top and bottom performing practices. In phase 2, patients with diabetes who were attributed to the aforementioned PCP practices were identified in a local health plan claims data base (a total of 3,221 patients), and the allowed amounts from their claims were used to evaluate differences in total and diabetes-related healthcare costs by providers’ performance.

**Results:**

Comparing 10,834 visits in high-performing practices to 8,235 visits in low-performing practices, referrals to certified diabetes care and education specialists and provider-to-provider electronic consults (e-consults) were higher in high-performing practices (Z = 6.06, *p* < .0001), while traditional referrals were higher in low-performing practices (Z = -6.94, *p* < .0001). The patient-to-provider ratio was higher in the low-performing group (M = 235.23) than in the high-performing group (M = 153.26) (Z = -2.82, *p* = .0048). Claims data analysis included 1,825 and 1,396 patients from high- and low-performing providers, respectively. The patient-to-provider ratio was again higher in the low-performing group (*p* = .009, V = 0.62). Patients receiving care from lower-performing practices were more likely to have had a diabetes-related hospital observation (5.7% vs. 3.9%, *p* = .02; V = 0.04) and higher diabetes-related care costs (*p* = .002; d = − 0.07); these differences by performance status persisted when controlling for differences in patient and physician characteristics. Patients seeing low-performing providers had higher Charlson Comorbidity Index scores (Mdn = 3) than those seeing high-performing providers (Mdn = 2).

**Conclusions:**

Referrals to the CDCES and e-Consult were associated with better measured diabetes outcomes, as were certain aspects of cost and types of hospital utilization. Higher patients to providers ratio and patients with more comorbidities were observed in low performing group.

**Supplementary Information:**

The online version contains supplementary material available at 10.1186/s12913-024-10745-2.

## Background

Although a growing number of patients with diabetes in the U.S. are meeting national quality metrics, only approximately 50% of these patients are able to achieve A1C < 7.0% [1]. A marked variability in diabetes outcomes at the local and national levels persists despite the availability of new effective treatments, better monitoring tools and increased awareness of the impact of diabetes on healthcare outcomes.

A patient’s unique risk profile and comorbidities may impact glycemic control, and these patient-specific factors likely lead to variation in outcomes among individuals with diabetes. However, the clinical course and outcomes of diabetes can be influenced by providers’ treatment approaches and management decisions, including diabetes and nutrition support, specialty referrals, medication choice, and frequency of follow-up visits [1]. Previous studies have demonstrated that dedicating resources to train primary care providers (PCPs) in evidence-based diabetes care is associated with positive patient outcomes [2]. However, little emphasis has been placed on identifying the specific treatment approaches or organizational support that may drive such improvements. To this end, the International Diabetes Federation (IDF) has called for research that explores the implementation of clinical guidelines to inform future best practices and policies in the setting of diabetes. Identifying the treatment approaches associated with positive diabetes outcomes is critical in allowing PCPs and healthcare delivery systems to improve the health of the populations they serve.

Additionally, in pursuit of the triple aim of health care—improved health and better patient experience at lower costs—there has been a great interest in measuring quality of care. The Centers for Medicare & Medicaid Services (CMS) and many healthcare organizations (i.e., National Committee for Quality Assurance [NCQA]) have proposed numerous metrics that align with the trend toward value-based care. Therefore, determining treatment approaches associated with positive diabetes outcomes, particularly those that confer consistent benefits across diverse settings and populations, is critical to achieve such goals. Considering that PCPs manage more than 90% of patients with diabetes, redesigned models of primary care have emphasized team-based diabetes treatment approaches and shared decision making [3–4].

The Allegheny Health Network (AHN), a multifacility academic healthcare system in Western Pennsylvania, has taken many steps toward value-based care to improve population health. To develop innovative treatment models for patients with diabetes, the AHN Division of Endocrinology partnered with the Primary Care Institute to monitor diabetes quality metrics and use these data to identify gaps in the diabetes-related care. In our academic healthcare system, we implemented a novel endocrinology compensation model that encourages collaboration between PCPs and endocrinologists through provider-to-provider electronic consults (e-consult), PCP education sessions, and regular visits to PCP practices by an endocrinologist and certified diabetes care and education specialist (CDCES) every 6 months to discuss practice-specific data and recent updates in diabetes management [5]. This initiative aims to standardize and improve diabetes care across the network while motivating endocrinologists to take on a supportive role for partnering in primary care services.

In this study, we sought to uncover gaps in treatment approaches that may guide the allocation of future resources and interventions. The objective was to identify treatment approaches that may contribute to the clinical outcomes of patients with diabetes in primary care settings, as well as variation in outcomes across practices within a large integrated health network. We used the Healthcare Effectiveness Data and Information Set (HEDIS), developed and maintained by the National Committee for Quality Assurance (NCQA), to rank primary care practices as high- or low-performing [6]. The primary outcome was to identify an association between individual treatment approaches and the achievement of established diabetes metrics (performance). In addition, we collected claims data of patients with diabetes managed by high- and low-performing practices to assess cost and other factors associated with potential variations in outcomes.

## Methods

This study includes two phases of data collection and analysis. In Phase 1, we assessed data directly from the electronic medical record (EMR) and survey data for clinics within a single provider network (Allegheny Health Network, AHN). Phase 2 supplemented these analyses with health insurance claims data for the patients assessed in Phase 1 who were members of Highmark Inc. health plans. The study protocol was reviewed by the AHN Institutional Review Board and was determined to be a clinical quality improvement project and not the human-subjects research, hence protocol was approved and requirement for informed consent was waived.

### Phase 1: treatment approaches

Inclusion and Exclusion Criteria of Participating Practices: All AHN primary care practices were included in the initial analysis, and 50 practices adopted HEDIS measures to monitor internal performance and quality improvement. HEDIS measures of all participating PCPs were collected using EMR and updated on a quarterly basis. Practices with fewer than 50 patients with diabetes reported per quarter were excluded. A total of 44 practices were measured against these metrics based on their performance over a one-year period that included the second, third and fourth quarters of 2019 and the first quarter of 2020. HEDIS metrics for diabetes were aggregated over the 12-month period and included in the analysis. Using the scoring system approved by NCQA, each practice was assigned a score (100 being the maximum score) and then ranked based on their score. The top 25% of practices (11 practices) were grouped as top-performing, and the bottom 25% of practices (11 practices) were grouped as low-performing, which comprised the sample of providers used in the analyses. These 22 practices had a total of 19,059 clinic visits by patients with a diagnosis of diabetes during the analysis period.

Treatment approaches: Five components of treatment approaches (variables) were collected using a combination of EMR and facility information. The 5 components included (1) the rate of traditional endocrine consults; (2) the rate of electronic provider to provider endocrine consults (e-consults), which is a service that is available to all practices included in the analysis; (3) the rate of CDCES referral orders (this service was equally available to all practices); (4) the utilization of insulin and noninsulin injectable medications (glucagon-like peptide-1 receptor agonists) among patients with diabetes; and (5) the PCP practice location and its proximity to the endocrine office (distance in miles) as it may impact treatment approaches, like referrals. Selection rates were based on the ratio of selected treatment approaches relative to PCP annual office visits of patients with diabetes. The 5 components chosen were based on their hypothesized effects on functional and clinical outcomes associated with diabetes management.

Data Analyses: Both descriptive and inferential statistics were computed for the treatment approaches. For continuous variables, t tests were conducted. For rate- and proportion-based variables (all referral- and medication-related variables), generalized linear models were used. Patients’ visit volume across performance status groups (ratio of patients to practitioners) was tested using a negative binomial model.

### Phase 2: insurance claims data

Data Source: The database used contained health plan claims for insurance products offered by Highmark Inc., an independent Blue Cross Blue Shield licensee. Both AHN and Highmark Inc. are owned by Highmark Health and are headquartered in Western Pennsylvania. Highmark Inc. offers independent, group, and Medicare Advantage health plans, and insured 5 + million members in Pennsylvania, Delaware, and West Virginia during the years of focus for analysis [7, 8].

Inclusion and Exclusion Criteria: We identified a total of 3,221 members with diabetes who were seen by the 78 physicians (MDs and DOs) that belonged to the top 11 and bottom 11 performing practices that were ranked in Phase 1. These members were identified using the criteria of (i) at least one approved claim with a primary diagnosis of diabetes (ICD 10: E08.xx, E09.xx, E10.xx, E11.xx, E12.xx, or E13.xx.) between April 1st, 2019, and March 31st, 2020, (ii) twelve months of continuous enrollment during the aforementioned timeframe, and (iii) receiving treatment from one of the aforementioned 78 AHN providers.

Member Characteristics, Physician Characteristics and Patient Load: Demographic and insurance plan types were obtained from members’ enrollment information. Insurance plan type was categorized as commercial (e.g., plans purchased individually or through participating employers), senior (e.g., Medicare Advantage), or other (e.g., federal employee). We defined patients’ health status using the Charlson Comorbidity Index (CCI), which quantifies long-term mortality in individuals with multiple comorbidities [9–10]. The CCI for each member was calculated using ICD-10 codes appearing on members’ claims from the study period. Type of diabetes diagnosis (type 1, type 2, secondary, both type 1 and type 2) and insulin use were derived from the presence of corresponding diagnosis codes and national drug codes (NDCs) in a member’s claims throughout the 12-month period. In addition to member characteristics, physician characteristics were extracted from standard information maintained within the claims database (e.g., age, sex, degree type). The patient-level data, along with linked practice/physician information from the claims, were used to calculate the number of patients seen within each practice and by each physician. Note that due to the nature of using claims data, these patient load estimates reflect the number of patients who are Highmark members and not the total number of patients.

Cost and Utilization: Allowed amounts (i.e., negotiated costs for service) were used to calculate the total cost of diabetes-related care and all other healthcare for the twelve-month study period. Diabetes-related cost of care was calculated as the sum of all claims costs that fell into one of the following categories: medical claims that had diabetes as the primary or admitting diagnosis (ICD 10 E08.xx – E13.xx), diabetes medication claims, or claims for diabetes-related durable medical equipment (DME), i.e., glucose monitoring supplies. All claims not meeting the diabetes-related criteria were summed to create the total cost of other care. Details on medications, DME, and CPTs used for categorization of diabetes-related spending and utilization are provided in the Supplementary Materials (eTables 1–3). Diabetes-related hospital utilization was identified using a combination of diagnosis codes, claim type codes (e.g., inpatient vs. professional claims), and CPTs from individual claims. Three types of diabetes-related hospital utilizations over the 12-month period were identified (coded as any vs. none per member): hospital observations, inpatient admissions, and emergency department use (eTable 3). Determination of hospitalizations as diabetes-related followed the same coding strategy described above for costs.

Data Analysis: Descriptive statistics were computed for patient and physician characteristics (e.g., demographics, type of diabetes diagnoses) as a function of performance status (high vs. low). Differences in patient and physician characteristics were evaluated using Wilcoxon rank sum tests, chi-squared tests, and Fisher’s exact tests as appropriate. Cohen’s d and Cramer’s V are provided to quantify the magnitude of performance group differences. Follow-up analyses to determine factors that may underlie differences in practices’ performance status consisted of comparisons of the number of physicians within practices, the number of patients seen per physician, patient’s health status (CCI scores), diabetes-related care costs, and diabetes-related hospital utilization (stratified by type). These patient and care-related factors were compared using Wilcoxon rank-sum tests (continuous variables) and Fisher’s exact tests (categorical variables).

Cost and utilization measures that differed by performance status were compared using logistic and quantile regression to evaluate differences while controlling for patient and physician factors. Quantile regression was selected to relax assumptions of normality and allow specification of the part(s) of the outcome distribution to predict using percentiles. For example, specifying the 50th percentile allows prediction of the median. Quantile regression was well designed for the cost data because the distribution was both skewed and bimodal. The percentiles chosen for modeling corresponded to the two modes for the high-performance group. In all models, the primary predictor of interest was performance status; control variables included patient age (centered), physician age (centered), patient sex, physician sex, plan type (commercial, senior, or other), CCI score, presence of type 1 diabetes diagnosis (yes/no), insulin use (any/none), and members’ spend on non-diabetes-related care (in $100 units). Cost and utilization measures of interest in these adjusted analyses had low correlations within physicians (intraclass correlation coefficients between 0.00 and 0.02), and as such analysis methods that allowed for modeling individual physician-level variance were not pursued (e.g., mixed effects modeling).

Analyses were conducted using RStudio Workbench (version 1.4.1717-3) running R version 3.6.3 with an alpha level of 0.05 for significance. Analyses, tables, and figures were created using the following packages: ggplot2, ggridges, effsize, rstatix, quantreg, marginaleffects, performance, and gtsummary [11–18].

## Results

### Phase 1: treatment approaches

Treatment approaches that differed between groups were the rate of CDCES referrals (Z = 6.06, *p* < .0001), e-consults (Z = 3.76, *p* = .0002), and traditional referrals (Z = − 6.94, *p* < .0001). CDCES referrals (M = 0.06) and e-consults (M = 0.006) were higher in the high-performing group than in the low-performing group (M = 0.04 for CDCES referrals, M = 0.0020 for e-consults). In contrast, the low-performing group (M = 0.08) had more traditional referrals than the high-performing group (M = 0.05). The ratio of patients to providers between groups was significantly different (Z = -2.82, *p* = .0048). The ratio of patients to providers for the low-performing group (M = 235.23) was higher than that of the high-performing group (M = 153.26). Tables 1a and 1b contain descriptive statistics and a comparison of treatment approaches between high- and low-performing groups.

**Table 1a:** Descriptive statistics and comparisons of treatment practice variables for the whole sample and as a function of performance status (high vs. low)

Treatment approach characteristics	Performance Status		*p*-value
	High	Low	
Total visits included in analysis	10,834	8235	0.2635
CDCES referral per 100 visits a year (*n*)	6.36	3.62	< 0.0001
E-consult referrals per 100 visits (*n*)	0.60	0.17	0.0002
Traditional referrals per 100 visit (*n*)	5.11	6.57	< 0.0001
% visits with insulin users	28	29	0.5392
% patients using other injectable medications	8	7	0.2157
Average proximity to Endocrine practice- miles	5.87	5.60	0.8909
Ratio of total number of visits/providers (APP and MD)	138.90	196.07	0.0515

**Table 1b:** Mean and SD differences between high- and low-performing groups in terms of treatment practice variables

Treatment approach characteristics	Performance Status				*p* value
	High		Low		
	Mean	Std	Mean	Std	
CDCES referral (*n*)	62.64	50.68	29.80	24.26	< 0.001
E-consult (*n*)	5.91	7.15	1.40	1.96	< 0.001
Traditional consult (*n*)	50.36	32.98	54.10	47.10	< 0.001
Insulin pattern (%)	0.28	0.03	0.29	0.05	0.576
Injectable pattern (%)	0.08	0.03	0.08	0.05	> 0.99
Proximity to endo office (miles)	5.87	4.17	5.60	5.00	0.891
Annual patient visits (*n*)	984.9	545.3	748.6	408.1	0.264

### Phase 2: insurance claims data

Most demographic and health characteristics of patients receiving care from high- versus low-performing practices were similar; in instances where they did differ, the effects were typically small (e.g., Cohen’s d < |0.18|). The largest of these differences was in comorbidities: patients seeing physicians at low-performing practices had higher Charlson Comorbidity Index (CCI) scores (Mdn = 3) than those seeing physicians at high-performing practices (Mdn = 2); details are provided in Table 2, and eFigure 2 depicts the distributions of CCI scores. The second largest difference was in plan type that patients were enrolled in; patients seeing physicians at high-performing practices were more likely to be enrolled in commercial (52% vs. 44% for high vs. low performing) and senior plans (6.5% vs. 4.9% for high vs. low performing). See Table 2 for details of tests performed and results of all comparisons.

**Table 2:** Descriptive statistics and comparisons of patient and practitioner characteristics of those receiving or providing care at the high- and low-performing practices, both overall and as a function of performance status

Sample/Characteristic	Overall ^1^	Performance Status		*p* value^2^	Effect size^3^
		High^1^	Low^1^		
Patients					
Number	3,221	1,825	1,396	-	-
Age	66 (58, 75)	65 (57, 74)	67 (59, 76)	< 0.001	-0.14
Sex				0.003	0.05
Female	1,484 (46%)	882 (48%)	602 (43%)		
Male	1,737 (54%)	943 (52%)	794 (57%)		
Plan Type				< 0.001	0.09
Commercial	1,553 (48%)	941 (52%)	612 (44%)		
Senior	187 (5.8%)	119 (6.5%)	68 (4.9%)		
Other	1,481 (46%)	765 (42%)	716 (51%)		
CCI	2 (1, 4)	2 (1, 4)	3 (2, 4)	< 0.001	-0.18
Taking Insulin				0.200	0.02
No	2,471 (77%)	1,415 (78%)	1,056 (76%)		
Yes	750 (23%)	410 (22%)	340 (24%)		
Diabetes Diagnoses^4^				0.300	0.04
T1	49 (1.5%)	32 (1.8%)	17 (1.2%)		
T2	2,923 (91%)	1,660 (91%)	1,263 (90%)		
T1/T2	238 (7.4%)	129 (7.1%)	109 (7.8%)		
Secondary	11 (0.3%)	4 (0.2%)	7 (0.5%)		
Practitioners					
Number	78	51	27	-	-
Age	47 (36, 55)	41 (34, 53)	53 (45, 65)	0.002	-0.83
Sex				0.022	0.23
Male	41 (53%)	22 (43%)	19 (70%)		
Female	37 (47%)	29 (57%)	8 (30%)		
Degree Type				0.300	0.09
DO	26 (33%)	19 (37%)	7 (26%)		
MD	52 (67%)	32 (63%)	20 (74%)		
Specialty				0.500	0.14
Family Practice	40 (51%)	27 (53%)	13 (48%)		
Internal Medicine	35 (45%)	23 (45%)	12 (44%)		
Other	3 (3.8%)	1 (2.0%)	2 (7.4%)		

^1^ Median (IQR); n (%)

^2^ Wilcoxon rank sum test; Pearson’s Chi-squared test; Fisher’s exact test. Hyphen (-) indicates no statistical comparison was performed.

^3^ Effect sizes are Cohen’s *d;* Cramer’s *V*

^4^ T1 and T2 refer to Type 1 Diabetes and Type 2 Diabetes, respectively

**Fig. 1:**
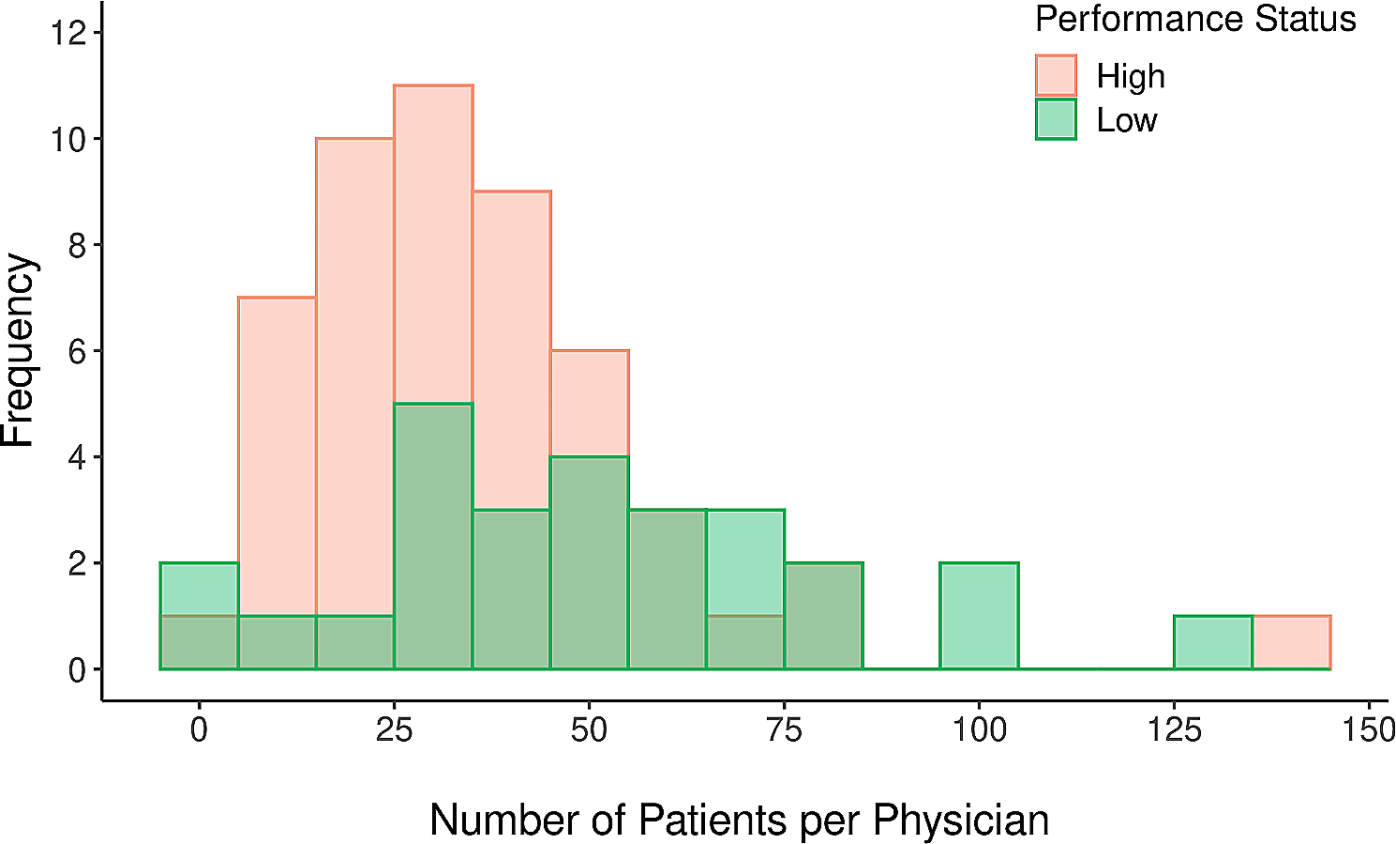
Frequency histogram of the number of patients with diabetes seen per physician who received diabetes-related care during the study period, with the color of bars corresponding to physicians within high (coral) versus low (green) performing practices

Physicians at low and high performing practices differed in respect to several demographic characteristics, as well as in considerations of patient load. Specifically, physicians at high performing practices were significantly younger (Mdn age = 41) than those at low-performing practices (Mdn age = 53), and the effect was large (*d* = -0.83; see Table 2 for details). There was also a significantly larger percentage of physicians identifying as female at high performing practices (57%) than at low performing practices (30%), although this effect was smaller. In agreement with the results of phase 1 analysis, physicians at low-performing practices had significantly higher patient loads (median = 52 patients per physician, Q1 = 32, Q3 = 72) than those at high-performing practices (median = 32 patients per physician, Q1 = 22, Q3 = 46; *p* = .009, V = 0.62). Figure 1 shows the distributions of patient counts per physician by performance status. Among low-performing practices, there were also significantly fewer physicians (high-performing median = 5, low-performing median = 3; *p* < .001, V = 0.95).

The unadjusted analyses of cost and utilization revealed several differences as a function of performance status, as shown in Table 3. However, the differences that did emerge were small: overall, those receiving care from lower-performing practices were more likely to have had a diabetes-related outpatient hospital observation (5.7% vs. 3.9%, *p* = .02; V = 0.04) and higher diabetes-related care costs (median difference = $552, *p* = .002; d = − 0.07). In follow-up analyses controlling for patient and physician characteristics, the difference in the likelihood of diabetes-related hospital observations as a function of performance status remained significant (*p* = .04; see Table 4). Visual analysis of the cost distribution indicated that the modes for the high-performance group were at values of $325 and $6,800, which corresponded to the 30th and 79th percentiles (see Fig. 1). The quantile regression predicting costs at these modal percentiles clarified that significant differences in cost were confined to the higher ends of the cost distribution. At the 79th percentile, low performance status was associated with increased diabetes care cost (b = $847.43, *p* = .01; see Table 4). In other words, the cost of diabetes care was similar across performance status groups at the lower end of the cost distribution; however, this changed as low-performing providers have higher costs at the high end of the distribution.

**Table 3:** Descriptive statistics and results of unadjusted comparisons of patients’ diabetes-related care costs and several types of hospital utilization across performance status groups

Characteristic	Overall *N* = 3,221^*1*^	Performance Status		*p* value^2^	Effect size^3^
		High *n* = 1,825^*1*^	Low *n* = 1,396^*1*^		
Diabetes-Related Costs	891 (296, 6,071)	722 (278, 5,831)	1,274 (319, 6,399)	0.002	-0.07
Inpatient Admission				0.600	0.01
None	3,154 (98%)	1,785 (98%)	1,369 (98%)		
One or more	67 (2.1%)	40 (2.2%)	27 (1.9%)		
Hospital Observation				0.018	0.04
None	3,069 (95%)	1,753 (96%)	1,316 (94%)		
One or more	152 (4.7%)	72 (3.9%)	80 (5.7%)		
Emergency Department Use				0.500	0.01
None	3,030 (94%)	1,721 (94%)	1,309 (94%)		
One or more	191 (5.9%)	104 (5.7%)	87 (6.2%)		

^1^ Median (IQR); n (%)

^2^ Wilcoxon rank sum test; Pearson’s Chi-squared test

^3^  Cohen’s *d;* Cramer’s *V*

**Table 4:** Coefficient/effect estimates in units of the outcome (dollars, probability) from models predicting diabetes-related healthcare costs (quantile regression) and whether a patient had any outpatient hospital observations (logistic regression) during the study period

	DM-Related Spend (30th Percentile)				DM-Related Spend (79th Percentile)				Outpatient Hospital Observation (Any)			
Predictor	Estimate	95% CI	t value	*p*	Estimate	95% CI	t value	*p*	Estimate	95% CI	z value	*p*
Intercept	$216.46	$177.75, $255.18	9.20	< 0.001	$4,493.82	$3832.01, $5155.63	11.17	< 0.001	-	-	-	-
Age (Centered)	-$3.36	$-5.00, $-1.71	-3.35	< 0.001	-$43.64	$-70.18, $-17.10	-2.70	0.01	0.000	−0.001, 0.00	−0.82	0.41
Sex (Male)	$12.47	$-15.03, $39.98	0.75	0.46	$24.89	$-469.40, $519.19	0.08	0.93	−0.010	−0.025, 0.004	−1.41	0.16
CCI Score	$19.62	$11.06, $28.17	3.77	< 0.001	$253.09	$117.12, $389.06	3.06	0.002	0.010	0.007, 0.013	5.89	< 0.001
Type 1 DM Diagnosis (Yes)	$723.58	$456.25, $990.90	4.45	< 0.001	$2,484.07	$1794.67, $3173.47	5.93	< 0.001	0.017	−0.008, 0.041	1.33	0.18
Taking Insulin (Yes)	$4,601.52	$4237.37, $4965.67	20.78	< 0.001	$9,791.59	$9012.58, $10570.59	20.67	< 0.001	0.045	0.024, 0.065	4.25	< 0.001
Total of Other Medical Costs	-$0.10	$-0.21, $0.02	-1.34	0.18	-$0.67	$-1.02, $-0.31	-3.11	0.002	0.000	0.00, 0.00	2.78	0.005
Insurance Type (Other)	-$29.98	$-70.72, $10.77	-1.21	0.23	-$4,311.87	$-5022.61, $-3601.13	-9.98	< 0.001	0.002	−0.038, 0.042	0.10	0.92
Insurance Type (Senior)	$21.13	$-20.68, $62.94	0.83	0.41	-$2,727.80	$-3503.86, $-1951.74	-5.78	< 0.001	−0.014	−0.035, 0.007	−1.31	0.19
PCP Age (Centered)	$0.86	$-0.46, $2.19	1.07	0.28	$15.33	$-10.25, $40.90	0.99	0.32	0.000	−0.001, 0.00	−0.81	0.42
PCP Sex (Male)	-$23.93	$-53.34, $5.48	-1.34	0.18	$240.40	$-315.41, $796.21	0.71	0.48	−0.007	−0.024, 0.01	−0.82	0.41
Performer Status (Low)	$21.24	$-13.65, $56.12	1.00	0.32	$847.43	$287.44, $1407.43	2.49	0.01	0.017	0.000, 0.034	2.02	0.04

Note: For models predicting spend, the intercept reflects patients who were female, patients who saw female physicians, were enrolled in a commercial plan, did not have any claims for insulin, had no claims with a diagnosis corresponding to Type 1 diabetes mellitus (DM), and patients who saw physicians classified as high performers.

## Discussion

In this study, we ranked primary care practices based on NCQA-HEDIS measures for diabetes outcomes and then analyzed treatment approaches to identify the practices that are associated with better outcomes. Our findings indicated that, relative to low-performing practices, high-performing practices had higher e-Consult utilization rates, while low-performing practices had higher traditional referral rates.

E-consults are asynchronous consultative communications between clinicians, during which a PCP typically asks an endocrinologist to review the patient’s records and answer specific questions related to the patient’s diabetes management or endocrine disorders using a note that is placed in the patient’s records. The use of e-consults has improved access to care by facilitating the timeliness of specialist input [19]. In contrast, in-person consultations require administrative assistance to schedule and consult completion depending on clinic access [20]. E-Consults not only provide timely access to specialist input but also likely reflect PCP engagement in network initiatives and openness to partner with specialists to coordinate care. In this study, low-performing practices placed more traditional (face-to-face) endocrine referrals than high-performing PCPs. It is plausible that such a higher frequency of traditional consults may not necessarily lead to completion of endocrine consult, considering administrative barriers as well as low engagement in the network’s initiatives or limited motivation to partner with specialists.

We observed in this study that high-performing PCPs also had higher rates of CDCES referrals. Appropriate diabetes management requires daily monitoring of blood sugars and changes in diet and activity level, which in turn demands a patient’s active, continuous role [21]. CDCES not only make themselves available to advise patients on self-management but also facilitate care coordination and support providers by reducing administrative tasks such as collecting and reporting data, especially in complex cases [22–23]. Studies have demonstrated dismal utilization of CDCES [22–24]. In this study, high-performing practices more frequently made use of external support in the form of diabetes education and nutrition referrals. Developing management algorithms that prompt CDCES referrals, as well as tracking such referrals, have been suggested as strategies to improve uptake [22–25].

Higher-performing practices had lower patient-to-provider ratios (derived from the ratio of total annual visits to number of providers). This may represent a significant factor impacting provider performance, as high patient volumes limit providers’ ability to invest time in lifestyle and behavior counseling [21]. Numerous studies have shown that high-volume surgeons have better patient outcomes, but this trend is unlikely to translate to the primary care setting [26]. Higher patient volume often forces PCPs to spend less time per patient and hence compromises quality of visit and focus on more acute issues during visits and follow-up (i.e., adjusting insulin doses in between visits based on blood sugar readings), thereby potentially affecting diabetes-related outcomes. In our network, low-performing practices were found to have relatively fewer providers per practice (eFigure 3), perhaps indicating suboptimal support staff, which could also impact patient care and outcomes. Low-performing PCPs may not have the requisite support to care for complex diabetic cases compared to larger practices with more providers. Future studies exploring the optimal size of patient panels for PCPs and support in practice would likely help improve the quality of diabetes care.

Using the CCI, a weighted index that considers the number and seriousness of co-occurring diseases and a widely used predictor of prognosis [9], we found a relatively higher index in the low-performing group. This finding indicates that patients in low-performing practices may have more comorbidities, thereby partially explaining the variation in outcomes. In the setting of diabetes, PCPs manage patients with more medications and co-occurring diseases than ever before, which has made it challenging to provide optimal care as “first contact” providers [27]. Studies have shown that the type, number and severity of comorbid conditions can cause patients to deprioritize diabetes self-management, a factor that might have been responsible for higher A1cs among these patients [28]. Moreover, according to ADA guidelines, less stringent A1c targets are recommended for appropriate patients who have comorbidities that decrease life expectancy. Therefore, the “low performing” PCPs in this study who manage patients with high CCI indexes might in fact be appropriately pursuing less stringent A1c targets in accordance with guidelines [29]. In this manner, it highlights the limitations of the HEDIS system that need to be acknowledged. Our results indicate that patients with diabetes attributed to low-performing providers had slightly higher diabetes-related costs and hospital observations. We cannot ignore the likely impact of a higher CCI index on this finding, illustrating the difficulty of uncovering ties between cost and outcomes.

There is an urgent need for health care leaders to identify cost-effective, evidence-based solutions that meet quality standards [22]. The importance of innovative ways to support PCPs (i.e., diabetes education and the use of e-consults) to reduce workload and improve overall performance should be emphasized. In addition, while moving toward pay-for-performance is a step in the right direction and using metrics will likely help coordinate the actions and behaviors of providers, we must be aware of the shortcomings and possible adverse effects of this approach [30].

## Limitations

We acknowledge the many limitations that surround research relying on Electronic Medical Records and claims data, such as incomplete data and limited capture of relevant information. In particular with our study, all data were extracted from a single health care system (Phase 1) and a single health insurance provider (Phase 2) that are headquartered in the same region of the United States (Western Pennsylvania). In addition to these geographical considerations, the sample in Phase 2 likely contains greater proportions of individuals who work for select employers within the region (i.e., offer Highmark coverage to their employees) and those who enroll in Medicare Advantage and other senior plans. In sum, it is possible these results may not generalize to healthcare systems and patient populations in disparate regions, share idiosyncrasies associated with enrollment in Highmark plans in particular, or to those who do not need, or cannot afford Medicare Advantage coverage.

In addition to considerations surrounding our data source, there are limitations of HEDIS measures in assessing diabetes care quality, which we have tried to highlight in conducting this study. The definition of control, as mentioned, may vary with respect to HbA1c goals in patients with comorbidities. Finally, our claims-based analyses helped clarify what factors at the patient level—and some limited characteristics at the physician level—are associated with higher and lower performance on outcomes, which afforded the ability to control for characteristics in select analyses. However, we were not able to adjust for certain patient characteristics that are known to impact diabetes outcomes such as socioeconomic status, access care and other barriers to care, etc.

## Conclusion

This study highlights treatment approaches that may improve diabetes care in the primary care setting, likely impacting outcomes and costs downstream. High-performing practices more frequently used e-consults than traditional consultations. PCPs in the high-performance group also placed more CDCES referrals. Our findings indicate that more support and coordination are needed among PCPs to care for patients with diabetes and that providers’ patient panels should be optimized to allow for the time and resources needed to care for this population. In addition, while the use of quality metrics seems to be essential in monitoring performance and moving away from the fee-for-service model, our results underscore that these metrics should account for complex cases, comorbidities, patient panels and available resources (such as diabetes education).

### Electronic supplementary material

Below is the link to the electronic supplementary material.

Supplementary Material 1

## Data Availability

The dataset used and/or analyzed during the current study is available from the corresponding author upon reasonable request.

## Abbreviations

HEDIS: Healthcare Effectiveness Data and Information Set
CDCES: Certified diabetes care and education specialist
E: Consults-Electronic Consults
AHN: Allegheny Health Network
PCP: Primary Care Providers
IDF: International Diabetes Federation
CMS: Center for Medicare and Medicaid Services
NCQA: National Committee for Quality Assurance
