# Supplementary material for: Treatment approaches and costs associated with diabetes clinical metrics as measured by Healthcare Effectiveness Data and Information Set (HEDIS)
Source: BMC Health Serv Res. 2024 Mar 26;24:375. doi: 10.1186/s12913-024-10745-2 (PMC10967116; doi:10.1186/s12913-024-10745-2)
Supplement: Supplementary file 1 — Supplementary Material 1 [file 12913_2024_10745_MOESM1_ESM.docx]

**Supplementary Materials for**

**Management practices and Cost Associated with Better Diabetes Related Metrics**

**List of Contents**

eTable 1. Durable medical equipment (DME) codes and corresponding descriptions for DME considered to be diabetes-related and used for categorization of claims/costs.

eTable 2. Names/descriptions of medications considered to be diabetes-related and used for categorization of claims/costs.

eTable 3. Qualifying values from claim fields and the corresponding logic for categorizing claims as indicating one of three types of hospital utilization: inpatient, emergency department, or outpatient hospital observation.

eFigure1. Density plot illustrating the distribution of total cost of diabetes-related care over the course of the 12-month study period, with vertical lines corresponding to the percentiles of the modes for those with high-performing physicians. Colors correspond to the range of costs for the specified percentiles of the distributions; a constant of 1 was added to costs prior to log transformation.

eFigure 2. Density histogram of CCI scores of patients whose primary care providers were classified as either high performing (coral) or low performing (green).

eFigure 3. Frequency histogram of the number of physicians within each of the participating practices that were categorized as either high performing (coral) or low performing (green).

eTable 1. Durable medical equipment (DME) codes and corresponding descriptions for items considered to be diabetes-related and used for categorization of claims/costs.

| HCPCS | Description |
| --- | --- |
| A4230 | INFUSION SET FOR EXTERNAL INSULIN PUMP, NON NEEDLE CANNULA TYPE |
| A4231 | INFUSION SET FOR EXTERNAL INSULIN PUMP, NEEDLE TYPE |
| A4232 | SYRINGE WITH NEEDLE FOR EXTERNAL INSULIN PUMP, STERILE, 3CC |
| A4244 | ALCOHOL OR PEROXIDE, PER PINT |
| A4245 | ALCOHOL WIPES, PER BOX (100) |
| A4252 | BLOOD KETONE TEST OR REAGENT STRIP, EACH |
| A4253 | BLOOD GLUCOSE TEST OR REAGENT STRIPS FOR HOME BLOOD GLUCOSE MONITOR. PER 50 |
| A4255 | PLATFORMS FOR HOME BLOOD GLUCOSE MONITOR, 50 PER BOX |
| A4256 | NORMAL, LOW AND HIGH CALIBRATOR SOLUTION/CHIPS |
| A4258 | SPRING-POWERED DEVICE FOR LANCET, EACH |
| A4259 | LANCETS, PER BOX OF 100 |
| A9274 | EXTERNAL AMBULATORY INSULIN DELIVERY SYSTEM, INCLUDES ALL SUPPLIES |
| A9275 | HOME GLUCOSE DISPOSABLE MONITOR, INCLUDES TEST STRIPS |
| A9276 | SENSOR; SUBCUTANEOUS, DISPOSABLE, FOR USE WITH INTERSTITIAL CONTINUOUS GLUCOSE |
| A9277 | TRANSMITTER FOR USE WITH INTERSTITIAL CONTINUOUS GLUCOSE MONITORING SYSTEM |
| A9278 | RECEIVER (MONITOR); EXTERNAL, FOR USE WITH INTERSTITIAL CGM |
| E0607 | HOME BLOOD GLUCOSE MONITOR |
| E2101 | BLOOD GLUCOSE MONITOR WITH INTEGRATED LANCING/BLOOD SAMPLE |
| K0553 | SUPPLY ALLOWANCE FOR THERAPEUTIC (CGM), INCLUDES ALL SUPPLIES |
| K0554 | RECEIVER, DEDICATED, FOR USE WITH THERAPEUTIC CGM SYSTEM |
| S1031 | NONINVASIVE CGM DEVICE, RENTAL, SENSOR and REPLACEMENT, AND DOWNLOAD TO MONITOR |
| S1034 | ARTIFICIAL PANCREAS DEVICE SYSTEM, INCLUDING CGM, BLOOD GLUCOSE DEVICE, INSULIN PUMP AND COMPUTER ALGORITHM THAT COMMUNICATES WITH ALL OF THE DEVICES |
| S1035 | SENSOR; SUBCUTANEOUS, DISPOSABLE, FOR USE WITH ARTIFICIAL PANCREAS DEVICE SYSTEM |
| S1036 | TRANSMITTER; EXTERNAL, 1 DAY SUPPLY |
| S1037 | RECEIVER (MONITOR); EXTERNAL, FOR USE WITH ARTIFICIAL PANCREAS DEVICE SYSTEM |

eTable 2. Names/descriptions of medications considered to be diabetes-related and used for categorization of claims/costs.

| **Medication name/description** | **Medication name/description** | **Medication name/description** |
| --- | --- | --- |
| 1ST TIER UNIFINE PENTI | HUMALOG MIX 50-50 KWIK | ONETOUCH DELICA PLUS L |
| ACARBOSE | HUMALOG MIX 75-25 | ONETOUCH FINEPOINT LAN |
| ACCU-CHEK | HUMALOG MIX 75-25 KWIK | ONETOUCH LANCETS |
| ACCU-CHEK AVIVA PLUS | HUMULIN 70/30 KWIKPEN | ONETOUCH ULTRA BLUE TE |
| ACCU-CHEK FASTCLIX LAN | HUMULIN 70-30 | ONETOUCH VERIO |
| ACCU-CHEK SOFTCLIX | HUMULIN N | ONGLYZA |
| ADMELOG | HUMULIN N KWIKPEN | OZEMPIC |
| ADMELOG SOLOSTAR | HUMULIN R | PEN NEEDLE |
| ALCOHOL PADS | HUMULIN R U-500 | PEN NEEDLES |
| ALCOHOL PREP PADS | HUMULIN R U-500 KWIKPE | PENTIPS |
| ALOGLIPTIN | INJECT EASE LANCETS | PIOGLITAZONE HCL |
| APIDRA | INSULIN ASPART FLEXPEN | PIOGLITAZONE-METFORMIN |
| APIDRA SOLOSTAR | INSULIN LISPRO | PRANDIN |
| AUTOSHIELD DUO PEN NEE | INSULIN LISPRO KWIKPEN | PRECISION XTRA |
| AUTOSHIELD PEN NEEDLE | INSULIN PEN NEEDLE | PRODIGY TWIST TOP LANC |
| AVANDIA | INSULIN SYRINGE | REPAGLINIDE |
| BAQSIMI | INSUPEN | RYBELSUS |
| BASAGLAR KWIKPEN U-100 | INTEGRA SYRINGE | SAFETYGLIDE INSULIN SY |
| BYDUREON BCISE | INVOKAMET | SAFETYGLIDE NEEDLE |
| BYDUREON PEN | INVOKAMET XR | SAFETY-LOK SAFETY SYRI |
| COMFORT EZ INSULIN SYR | INVOKANA | SAFETY-LOK SYRINGES |
| COMFORT EZ PEN NEEDLE | JANUMET | SOLIQUA 100-33 |
| CONTOUR NEXT TEST STRI | JANUMET XR | STEGLATRO |
| CONTOUR TEST STRIP | JANUVIA | SURE COMFORT |
| DEXCOM G5-G4 SENSOR | JARDIANCE | SURE COMFORT LANCETS |
| DEXCOM G6 | JENTADUETO | SYNJARDY |
| EASY COMFORT | KETO-DIASTIX REAGENT | SYNJARDY XR |
| EASY COMFORT PEN NEEDL | KOMBIGLYZE XR | SYRINGE |
| EASY TOUCH | LANCETS | TOUJEO MAX SOLOSTAR |
| EASY TOUCH PEN NEEDLE | LANTUS | TOUJEO SOLOSTAR |
| ECLIPSE SYRINGE-NEEDLE | LANTUS SOLOSTAR | TRADJENTA |
| FARXIGA | LEVEMIR | TRESIBA FLEXTOUCH U-10 |
| FIASP | LEVEMIR FLEXTOUCH | TRESIBA FLEXTOUCH U-20 |
| FIASP FLEXTOUCH | METFORMIN ER OSMOTIC | TRUE METRIX GLUCOSE TE |
| FREESTYLE INSULINX TES | METFORMIN HCL | TRUEPLUS INSULIN SYRIN |
| FREESTYLE LANCETS | METFORMIN HCL ER | TRUEPLUS LANCETS |
| FREESTYLE LIBRE 14 DAY | MICROLET | TRUEPLUS PEN NEEDLE |
| FREESTYLE LITE TEST ST | MINI ULTRA-THIN II | TRUETRACK TEST STRIP |
|  |  | *Table continues* |
|  |  |  |
| **Medication name/description** | **Medication name/description** | **Medication name/description** |
| FREESTYLE PRECISION NE | MONOJECT INSULIN SYRIN | TRULICITY |
| FREESTYLE TEST STRIPS | NANO 2ND GEN PEN NEEDL | ULTICARE PEN NEEDLE |
| GLIMEPIRIDE | NATEGLINIDE | ULTRACARE PEN NEEDLE |
| GLIPIZIDE | NOVOFINE 32 | ULTRA-FINE MICRO PEN N |
| GLIPIZIDE ER | NOVOFINE AUTOCOVER | ULTRA-FINE MINI PEN NE |
| GLIPIZIDE XL | NOVOFINE PLUS | ULTRA-FINE NANO PEN NE |
| GLIPIZIDE-METFORMIN | NOVOLIN 70-30 | ULTRA-FINE ORIGINAL PE |
| GLUCAGEN | NOVOLIN 70-30 FLEXPEN | ULTRA-FINE SHORT PEN N |
| GLUCAGON EMERGENCY KIT | NOVOLIN N | UNIFINE PENTIPS |
| GLUMETZA | NOVOLIN R | UNIFINE PENTIPS PLUS |
| GLYBURIDE | NOVOLOG | VEO INSULIN SYRINGE |
| GLYBURIDE-METFORMIN HC | NOVOLOG FLEXPEN | V-GO 20 |
| GLYXAMBI | NOVOLOG MIX 70-30 | V-GO 30 |
| HUMALOG | NOVOLOG MIX 70-30 FLEX | VICTOZA 2-PAK |
| HUMALOG KWIKPEN U-100 | NOVOTWIST | VICTOZA 3-PAK |
| HUMALOG KWIKPEN U-200 | OMNIPOD | XIGDUO XR |
| HUMALOG MIX 50-50 | ONETOUCH DELICA | XULTOPHY 100-3.6 |

*Note*. The full list of medications by national drug code (NDC) contained more than 500 rows. As such, only unique names/descriptions are given for brevity; the complete list with NDCs is available on request from the corresponding author.

eTable 3. Qualifying values from claim fields and the corresponding logic for categorizing claims as indicating one of three types of hospital utilization: inpatient, emergency department, or outpatient hospital observation.

|  | Claim Fields | | | | Categorization Logic |
| --- | --- | --- | --- | --- | --- |
| Hospitalization Type | Claim Type | CPT/HCPCs  Codes | Place of Service | Other Indicators |  |
| Emergency Department | Outpatient OR Major Medical OR Professional | 99281-99285 | - | Emergency Room (ER) indicator* equal to a value of Y (yes) | Qualifying claim type and at least one of:   - Relevant CPT - ER indicator |
| Inpatient Admission | Inpatient | - | - | - | Any inpatient claim |
| Outpatient Observation | Outpatient OR Major Medical OR Professional | 99217,  99218-99220,  99221-99223,  99224-99226,  99231-99233,  99234-99236,  99238-99239,  99251-99255,  99291-99292,  G0378, G0379 | Ambulatory surgical center,  Off campus outpatient hospital,  On campus outpatient hospital,  Inpatient hospital,  Comprehensive outpatient rehabilitation facility | - | Claim must have a relevant value in each field listed |

*Note*. After categorization of claims using the above criteria, they were further classified as either being diabetes-related or not using the criteria described in the main text of the paper. For claims involving visits that spanned more than one day, only characteristics of the claims from the day of admission/first day were used. *Value is based on a combination of claim type and revenue codes that collectively capture emergency services provided in various settings.


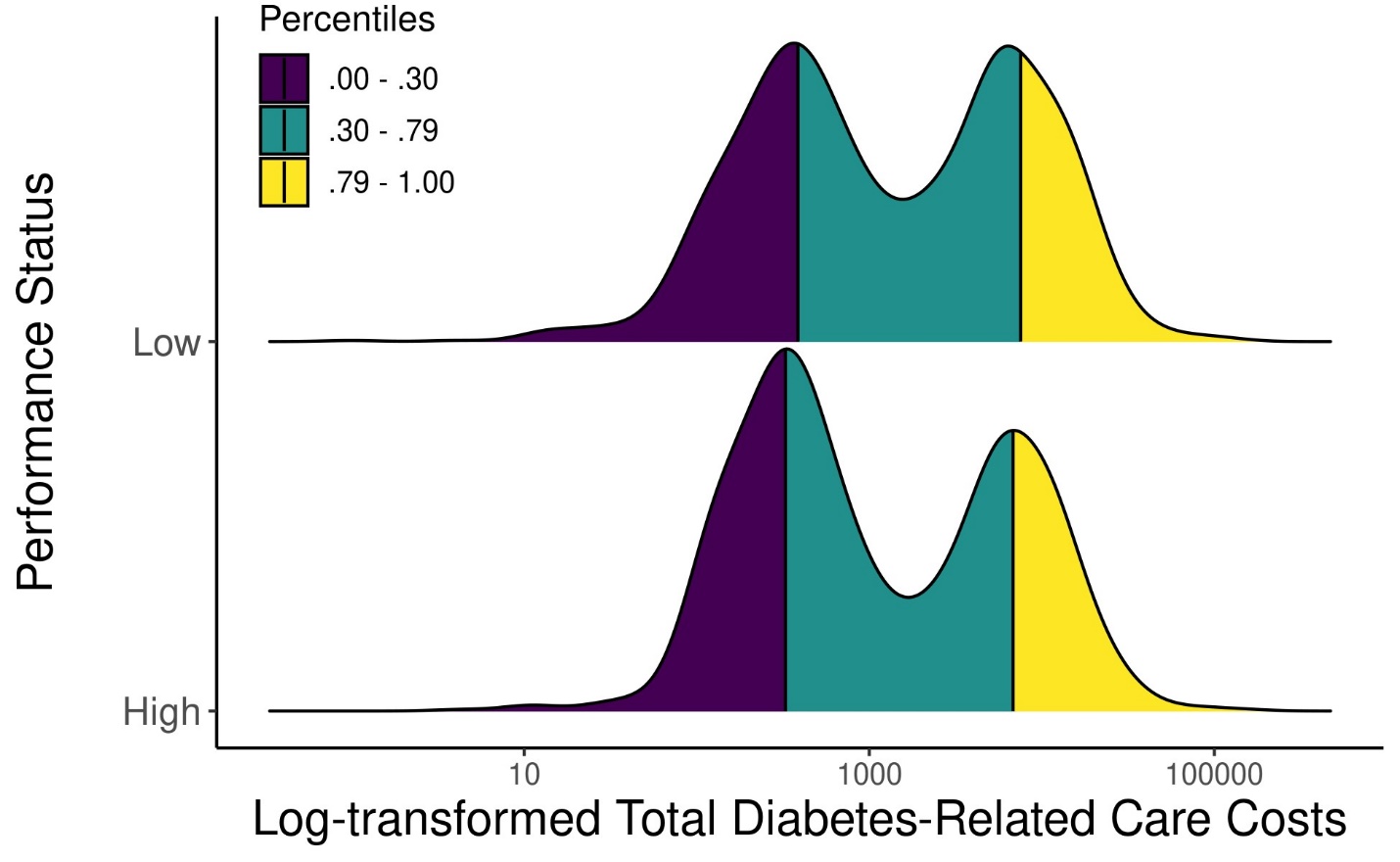


eFigure1. Density plot illustrating the distribution of total cost of diabetes-related care over the course of the 12-month study period, with vertical lines corresponding to the percentiles of the modes for those with high-performing physicians. Colors correspond to the range of costs for the specified percentiles of the distributions; a constant of 1 was added to costs prior to log transformation.


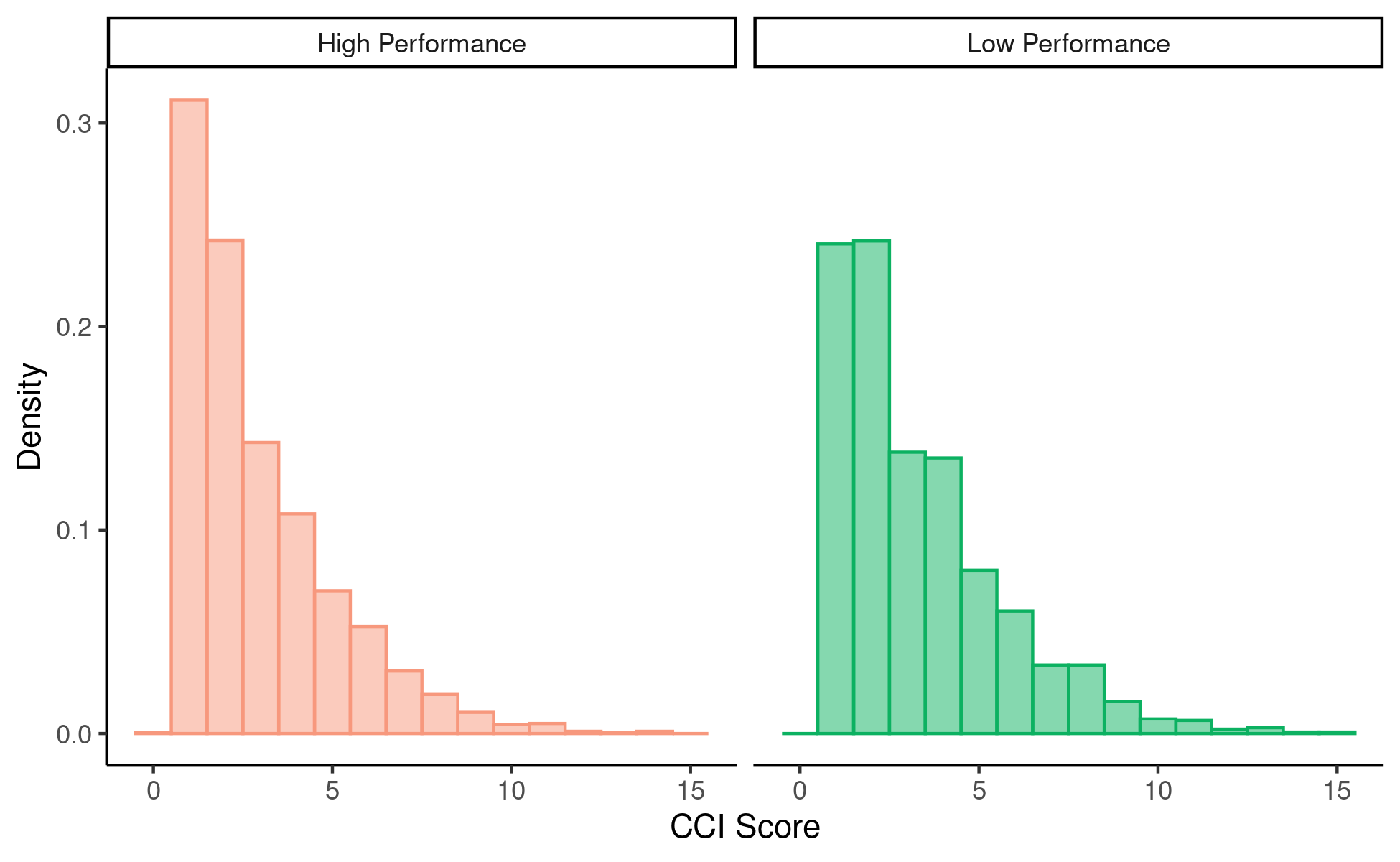
 eFigure 2. Density histogram of CCI scores of patients whose primary care providers were classified as either high performing (coral) or low performing (green).


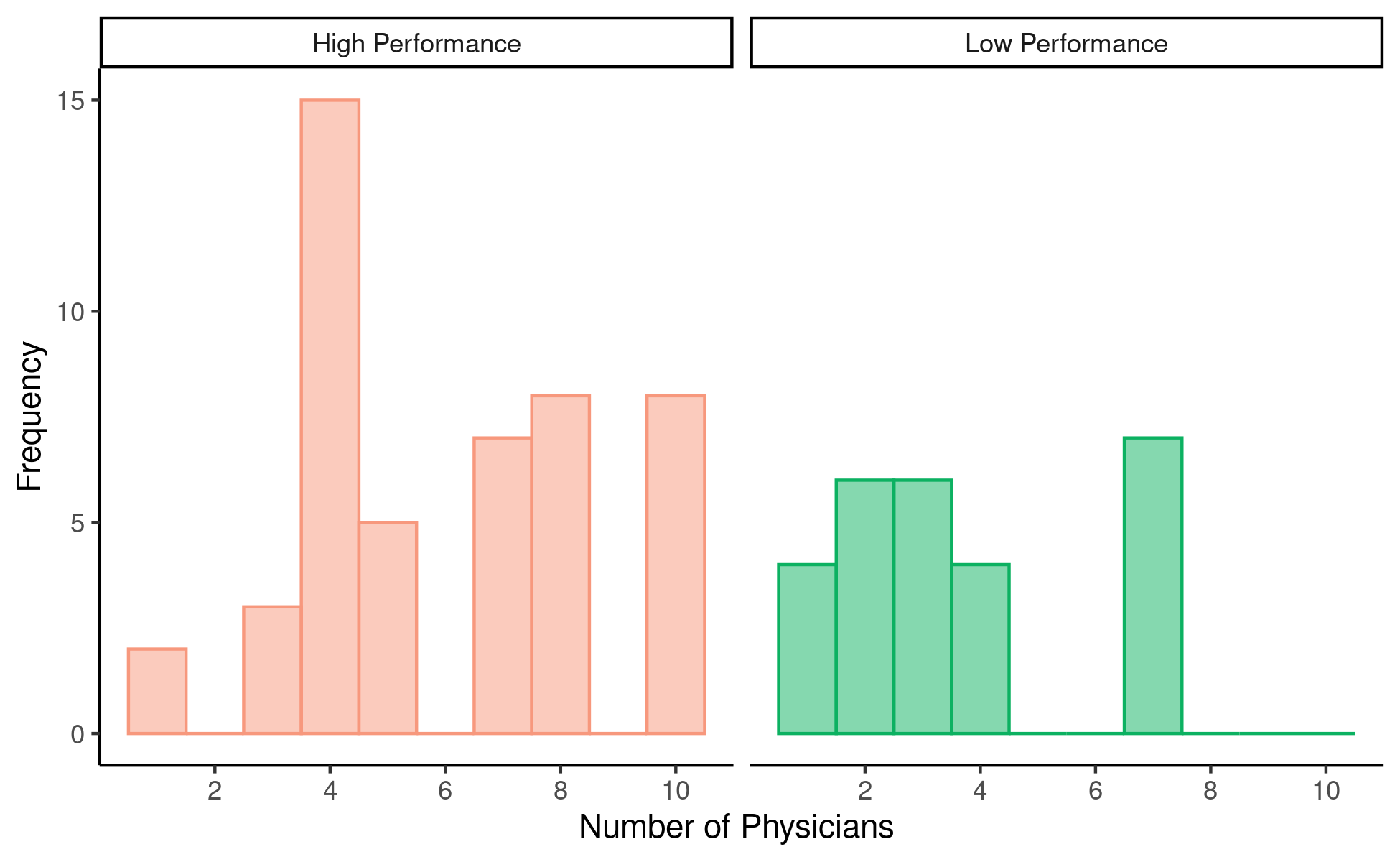


eFigure 3. Frequency histogram of the number of physicians within each of the participating practices that were categorized as either high performing (coral) or low performing (green).
